# A Case of Sclerosing Mesenteritis during Etanercept Therapy for Psoriatic Arthritis

**DOI:** 10.1155/2020/8899658

**Published:** 2020-07-11

**Authors:** Aleksandra Bukiej

**Affiliations:** Division of Rheumatology, Rush University Medical Center, 1611 W Harrison Street, Suite 510, Chicago, IL 60612, USA

## Abstract

Sclerosing mesenteritis (SM) is a chronic nonspecific mesenteric inflammation. I report a case of a 72-year-old male treated with etanercept for psoriatic arthritis for 7 years who developed abdominal discomfort, urinary retention, acute kidney injury, and bilateral ureteric obstruction. CT abdomen revealed retroperitoneal mass. Biopsy showed sclerosing mesenteritis. One year later, after discontinuation of etanercept, CT abdomen showed regression of the mass. To my knowledge, this is first case report of reversible sclerosing mesenteritis associated with etanercept therapy.

## 1. Introduction

Sclerosing mesenteritis is a chronic, nonspecific, inflammatory, and fibrotic process involving the small bowel mesentery with varying degrees of fibrosis, inflammation, and fat necrosis [1]. To my knowledge, this is first case report of sclerosing mesenteritis associated with etanercept therapy.

## 2. Case Report

A 72-year-old man presented with a two-month history of weight loss, worsening abdominal discomfort, fatigue, and acute onset of oliguria.

The patient has had a history of psoriasis for forty years treated with topical steroids and tacrolimus. Eight years prior to this event, he was diagnosed with psoriatic arthritis with peripheral involvement. He was first treated with naproxen followed by the addition of methotrexate during the next 8 months. Because of liver toxicity, methotrexate was discontinued. Etanercept 50 mg weekly was given for the next 7 years, and his disease was well controlled.

**Figure 1:**
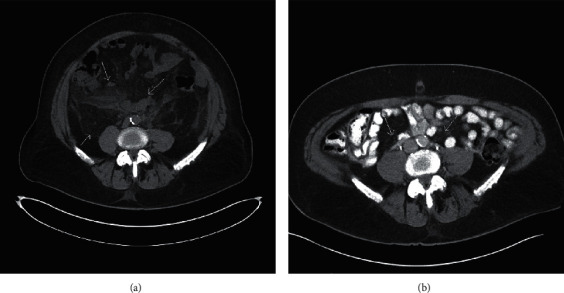
CT scan abdomen. (a) Initial and (b) follow-up scan 1 year later after discontinuation of etanercept, without systemic steroids.

**Figure 2:**
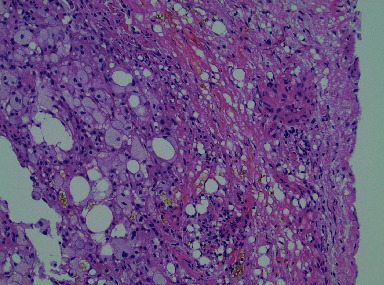
Histopathology of the mass: mesenteric inflammation with fibrosis and lipodystrophy (staining: hematoxylin-eosin; magnification: 20x).

He was also treated with allopurinol and probenecid for gout and with losartan for hypertension. On examination, he had bilateral flank tenderness, and the rest of the examination was unremarkable. His lab investigations showed Hg 11.5 gm/dl, WBC 8.29 per microliter, platelets 312 per microliter, urea 76 mg/dl, and creatinine 12.4 mg/dl. Urine examination was unremarkable and negative for Bence Jones protein. The C-reactive protein (CRP) was 100 mg/l. A computed tomography (CT) scan revealed diffuse hyperattenuation throughout the retroperitoneum and mesentery and bilateral mild hydronephrosis, with dilated ureters proximally thought to be due to retroperitoneal fibrosis (Figure 1). Bilateral ureteric stenting was performed resulting in good urine output, the kidney function improved, and repeat kidney parameters were normal. A laparoscopic biopsy was performed, which showed nonspecific inflammatory changes without granuloma or neoplastic cells (Figure 2). Immunoglobulin class 4 in the serum was normal. Etanercept was stopped, and a systemic steroid, prednisone 1 mg/kg, was recommended. The patient refused treatment with prednisone due to concern for side effects. Despite this, one year later, his CT scan showed marked improvement in the retroperitoneal mass (Figure 1). The patient denied arthralgias, and his inflammatory markers were normal as was his serum creatinine.

## 3. Discussion

Sclerosing mesenteritis (SM) is a part of a spectrum (including mesenteric lipodystrophy and mesenteric panniculitis) of idiopathic primary inflammatory and fibrotic processes that effect the mesentery. The clinical presentation usually includes abdominal pain, nausea, and vomiting. This process also may lead to abdominal masses [1]. The patient presented with abdominal discomfort, urinary retention, and acute kidney injury secondary to bilateral ureteric obstruction which is a more typical presentation for retroperitoneal fibrosis (RPF) [2]. In this case, RPF was also suggested by CT abdomen.

Famularo et al. [3] described five cases of RPF associated with psoriasis. In every case, RPF was followed by psoriatic flare and required systemic steroid treatment. In this case, the retroperitoneal mass was not preceded by a psoriatic flare. The patient has had psoriasis for 40 years treated with topical steroids and tacrolimus. Seven years before he developed SM, he was diagnosed with psoriatic arthritis, and etanercept was started. Moreover, three years before he started etanercept, the patient had MRI lumbar spine to assess axial involvement, and there was no evidence for retroperitoneal mass. Schiffmann et al. reported mesenteric panniculitis in the setting of cat-scratch disease during etanercept therapy for psoriatic arthritis [4]. They suggested that etanercept may have contributed to the splenic and mesenteric involvement. Couderc et al. described three cases of retroperitoneal fibrosis during etanercept therapy for rheumatoid arthritis [5]. My patient's symptoms and findings on CT abdomen were more suggestive for RPF. Biopsy showed nonspecific inflammation and evidence mostly like SM. The etiology of SM and RPF is unknown. It could be the same process, but in different stages or both forms of nonspecific inflammation. Etanercept use appears to have triggered some inflammation in the mesentery that, then, spread to the retroperitoneal space resulting in the patient's retroperitoneal mass and clinical symptoms from obstruction of the ureters. The treatment for SM includes corticosteroids and immunosuppressive therapy with azathioprine cyclophosphamide. My patient refused treatment with steroids. Given three cases of retroperitoneal fibrosis described by Couderc et al. [5] in patients treated with etanercept for rheumatoid arthritis, in my patient, there was suspicious for the association of etanercept and SM. Etanercept was discontinued. Despite the lack of treatment for SM, repeated CT abdomen showed progressive regression of the retroperitoneal mass and normalization of inflammatory markers.

Catanoso et al. reported the case of a woman with idiopathic RPF treated with infliximab, a monoclonal antibody directed against TNF alpha [6]. In contrast, I have described the patient who developed retroperitoneal mass while receiving etanercept for psoriatic arthritis. Verhoeven et al. reported a case of aortitis during etanercept therapy for ankylosing spondylitis [7]. The use of TNF alpha blockers has been linked with the paradoxical development of systemic and organ-specific autoimmune processes [7, 8].

A unique feature in my case is the development of SM during etanercept therapy. After etanercept was discontinued, the regression of retroperitoneal mass was observed. Moreover, the first line treatment for SM includes systemic steroids, but my patient refused steroids, and despite that, there was radiological and clinical improvement with an associated decrease in inflammatory markers. Such a case raises strong suspicion for a causative role of etanercept in the occurrence of this patient's retroperitoneal mass and could, therefore, represent a new potential adverse event.
